# Caffeic Acid Phenethyl Ester Reduces Ischemia-Induced Kidney Mitochondrial Injury in Rats

**DOI:** 10.1155/2017/1697018

**Published:** 2017-08-13

**Authors:** Sonata Trumbeckaite, Neringa Pauziene, Darius Trumbeckas, Mindaugas Jievaltas, Rasa Baniene

**Affiliations:** ^1^Neuroscience Institute, Lithuanian University of Health Sciences, Eiveniu Str. 4, LT-50161 Kaunas, Lithuania; ^2^Department of Pharmacognosy, Medical Academy, Lithuanian University of Health Sciences, Eiveniu Str. 13, LT-50166 Kaunas, Lithuania; ^3^Institute of Anatomy, Lithuanian University of Health Sciences, Mickeviciaus Str. 9, LT-44307 Kaunas, Lithuania; ^4^Department of Urology, Medical Academy, Lithuanian University of Health Sciences, Eivenių g. 2, LT-50161 Kaunas, Lithuania; ^5^Department of Biochemistry, Medical Academy, Lithuanian University of Health Sciences, Eiveniu Str. 4, LT-50161 Kaunas, Lithuania

## Abstract

During partial nephrectomy, the avoidance of ischemic renal damage is extremely important as duration of renal artery clamping (i.e., ischemia) influences postoperative kidney function. Mitochondria (main producer of ATP in the cell) are very sensitive to ischemia and undergo damage during oxidative stress. Finding of a compound which diminishes ischemic injury to kidney is of great importance. Caffeic acid phenethyl ester (CAPE), biologically active compound of propolis, might be one of the promising therapeutic agents against ischemia-caused damage. Despite wide range of biological activities of CAPE, detailed biochemical mechanisms of its action at the level of mitochondria during ischemia are poorly described and need to be investigated. We investigated if CAPE (22 mg/kg and 34 mg/kg, injected intraperitoneally) has protective effects against short (20 min) and longer time (40 min) rat kidney ischemia in an *in vitro* ischemia model. CAPE ameliorates in part ischemia-induced renal mitochondrial injury, improves oxidative phosphorylation with complex I-dependent substrate glutamate/malate, increases Ca^2+^ uptake by mitochondria, blocks ischemia-induced caspase-3 activation, and protects kidney cells from ischemia-induced necrosis. The protective effects on mitochondrial respiration rates were seen after shorter (20 min) time of ischemia whereas reduction of apotosis and necrosis and increase in Ca^2+^ uptake were revealed after both, shorter and longer time of ischemia.

## 1. Introduction

Kidney ischemia-reperfusion (I/R) injury is characterized by restriction of blood supply to an organ followed by restoration of blood flow and reoxygenation. Kidney injury may occur after infarction and sepsis, during partial nephrectomy and a surgical procedure, and when kidney tumor is removed after clamping renal artery [1]. Clamping time (i.e., duration of ischemia) is thought to be a major factor in determining postoperative kidney dysfunction. During partial nephrectomy, the avoidance of ischemic renal damage is extremely important as duration of renal artery clamping influences postoperative kidney function. It is well described that mitochondria are very sensitive to ischemia-induced injury and undergo damage during oxidative stress [2]. Impairment in Ca^2+^ homeostasis, formation of reactive oxygen species (ROS), release of proapoptotic proteins, and loss of ATP synthesis occur during ischemia [3], and all these processes might lead to cell death in the form of apoptosis or necrosis. Recently, when more and more partial kidney resections are performed for bigger kidney tumors, the time of ischemia is extremely important for postoperative kidney function. Completion of open partial nephrectomy with 30 minutes renal artery clamping is generally easily achieved in standard T1 stage renal tumor, but longer ischemia time is necessary in bigger tumors or in tumors of unfavorable localization [4]. This can be achieved using cold ischemia when kidney can tolerate ischemia up to two hours [5]. However, cooling of kidney during laparoscopic procedure is technically complicated and so rarely used. Therefore, there is a need of anti-ischemic agents in situations when longer time of kidney clamping is necessary. For improving ischemia tolerance, much attention has focused on new antioxidants or free radical scavengers with high potency, easy permeability to cellular compartments, and low toxicity. Caffeic acid phenethyl ester (CAPE) due to high lipophilicity might be one of the promising therapeutic agents against I/R-caused damage. Finding of a biologically active compound which diminishes negative ischemia impact to kidney function would be a solution in situations when longer time of kidney clamping is necessary.

CAPE is one of the most active compounds of propolis, exhibiting wide range of biological properties. CAPE possesses antioxidant, anti-inflammatory, and anticancer activity and regulates apoptosis [6, 7]. It has been demonstrated that CAPE (10 *μ*mol/kg/day for 11 days) prevents cyclosporine A and lipid peroxidation-mediated nephrotoxicity via inhibition of oxidative process [8]. Another study showed that pretreatment with intraperitoneal CAPE (10 *μ*mol/kg/day) protects kidney from ischemia/reperfusion injury [9] by partial inhibition of neutrophil sequestration into the kidney. In contrast, Roso et al. state [10] that CAPE (10 *μ*mol/kg/day) demonstrated greater functional and anatomic renal injury during ischemia and reperfusion in rats anesthetized with isoflurane [10] and no beneficial CAPE effect in the glycerol-induced acute renal failure model [11]. Wei et al. showed that intraperitoneal injections of CAPE (40 mg/kg/day) protected hypoxic ischemia-induced neonatal rat brain damage by inhibiting caspase-3 activation, expression of inducible nitric oxide synthase, and Ca^2^^+^-induced cytochrome *c* release [12]. Khan et al. observed that CAPE (1–10 mg/kg) protected the brain from ischemia-reperfusion-induced injury, increased nitric oxide and glutathione levels, and decreased lipid peroxidation [13]. Parlakpinar et al. indicated that CAPE (50 *μ*mol/kg) had protective effect against cardiac ischemia-reperfusion-induced apoptosis and acts in the heart as scavenger of free radicals [14]. Despite all these controversial data, detailed biochemical mechanisms at the level of mitochondria during ischemia/reperfusion are poorly described and need to be investigated.

Thus, the aim of this study was to test our hypothesis if caffeic acid phenethyl ester (CAPE) may protect kidney mitochondria from ischemic injury.

## 2. Materials and Methods

### 2.1. Animals and Experimental Model

The experimental procedures used in the present study were performed according to the permission of the Lithuanian Committee of Good Laboratory Animal Use Practice (number 0228/2012). Adult male Wistar rats weighing 200–250 g were housed under standard laboratory conditions and maintained on natural light and dark cycle and had free access to food and water. Animals were acclimatized to laboratory conditions before the experiment. Animals were pretreated with two doses (22 mg/kg and 34 mg/kg) of intraperitoneal injections of CAPE 1.5 h prior induction of ischemia. Then, animals were sacrificed and the kidneys were removed, washed free of blood in warm (37°C) 0.9% KCl solution, placed in a humidified chamber maintained at 37°C, and were exposed for 20 min, 40 min of total (*in vitro*) ischemia. After that time, kidney tissue was used for isolation of mitochondria.

### 2.2. Chemicals

Succinic acid, glutamic acid, cytochrome c from bovine heart, adenosine-5'-diphosphate sodium salt (ADP), CAPE, malic acid, KH_2_PO_4_, ethylene glycol-bis-(b-aminoethylether)-N,N,N',N'-tetraacetic acid (EGTA), ethylenediamine tetraacetic acid (EDTA), Tris, amytal, and atractyloside were obtained from “Sigma.” Mannitol, sucrose, KCl, HEPES, and MgCl_2_ were obtained from “Roth.”

### 2.3. Preparation of Renal Mitochondria

Kidney tissue was cut into small pieces and homogenized in the medium containing 250 mM sucrose, 10 mM Tris-HCl, and 1 mM EDTA (pH 7.3). Cytosolic and mitochondrial fractions were separated by differential centrifugation (5 min at 750 ×g and 10 min at 10,000 ×g, two times), and pellet was suspended in an isolation medium.

### 2.4. Measurement of Mitochondrial Respiration

Mitochondrial respiration (oxygen consumption) rate was measured at 37°C using Clark-type electrode in 1.5 ml incubation medium containing 150 mM KCl, 10 mM Tris-HCl, 5 mM KH_2_PO_4_, and 1 mM MgCl_2_ × 6H_2_O, pH 7.2. The mitochondrial leak respiration (*V*_0_) was recorded in the medium supplemented with mitochondria and respiratory substrates: complex I dependent (5 mM glutamate + 5 mM malate) or complex II dependent (15 mM succinate + 2 mM amytal) but without ADP. Glutamate dehydrogenase oxidizes glutamate to *α*-ketoglutarate, and in this reaction, NAD^+^ is reduced to NADH (NADH is a substrate for complex I of mitochondrial respiratory chain). Oxidation of succinate is coupled with reduction of FAD to FADH_2_ (FADH_2_ is a substrate for complex II of mitochondrial respiratory chain). Then, excess of ADP (1 mM) was added in order to measure the state 3 respiration rate (*V*_3_). After addition of cytochrome c, respiration rate *V*_3_ + cyt c was registered. The increase in *V*_3_ + cyt c represents the damage of mitochondrial outer membrane and release of cytochrome c. Nonphosphorylating respiration rate (*V*_ATR_) was measured in the presence of excess of atractyloside (0.12 mM) in order to inhibit ATP/ADP translocator and to block ATP synthesis.

### 2.5. Measurement of Complex I Activity

Mitochondria immediately after isolation were freeze-thawed four times. Complex I activity was determined spectrophotometrically by following the kinetics of NADH oxidation at 340 nm, in the medium containing 10 mM KH_2_PO_4_ (pH 8.0), 1 mg/ml Antimycin A, 0.1 mM NADH, 100 mM coenzyme Q_1_, and 0.05 mg/ml fractured mitochondria. Rotenone-sensitive NADH oxidation rate was recorded in the presence of 10 *μ*mol of rotenone. Complex I activity was calculated as the difference between NADH oxidation rate without/with rotenone using the NADH extinction coefficient 6.22 M^−1^ cm^−1^.

### 2.6. Measurement of Mitochondrial Calcium Uptake Capacity

Mitochondrial calcium uptake capacity was measured fluorimetrically (at 37°C) with Calcium Green-5N (excitation at 506 nm, emission at 535 nm) in medium containing 200 mM sucrose, 1 mM KH_2_PO_4_, 10 mM Tris-HCl, 10 *μ*M EGTA, 0.3 mM pyruvate plus 0.3 mM malate, pH 7.4, and 0.05 mg/ml of mitochondrial protein as described previously [15]. For calibration of the signal, known amounts of CaCl_2_ (100 *μ*M) were added. Then, CaCl_2_ (100 *μ*M) was added in two-minute intervals until opening of permeability transition pore occurred.

### 2.7. Measurement of Caspase Activity

Postmitochondrial supernatant was additionally centrifuged for 30 min at 10000 ×g, and the resulting supernatant was used for determination of caspase activity. For measurement of caspase-3-like activity, 1 mg/ml of total cytosolic protein was incubated for 60 min in buffer containing 250 mM sucrose, 5 mM HEPES, 2 mM EGTA (pH 7.3 at 37°C), and 0.1 mM acetyl-Asp-Glu-Val-Asp-7-amido-4-methylcoumarin (DEVD). The hydrolysis of caspase substrate was followed fluorimetrically, excitation was set at 380 nm, and emission at 460 nm. Substrate cleaving activity was completely suppressed by 0.02 mM N-acetyl-Asp-Glu-Val-Asp-aldehyde, a reversible inhibitor of caspase-3.

### 2.8. Electron Microscopy

The control and ischemic samples of 1-2 × 2-3 mm from the kidneys were transferred to the fixative buffer containing 2.5% glutaraldehyde in 0.1 M phosphate buffer (pH 7.4). The taken samples were stored in fixative for at least 4 h at room temperature or overnight at 4°C and analyzed as described in [15].

### 2.9. Statistical Analysis

Data are presented as mean ± SEM of 4 separate experiments. The mean for individual experiment was obtained from at least three repetitive measurements. Statistical analysis was performed using the software package SPSS version 16.0 for Windows.

## 3. Results

### 3.1. Effect of CAPE on Ischemia-Induced Mitochondrial Injury

**Figure 1:**
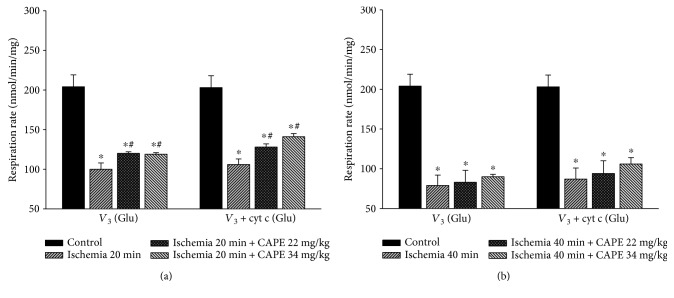
Effects of ischemia on renal mitochondrial state 3 respiration rate with glutamate/malate as substrates. Mitochondrial respiration rate was measured as described in Materials and Methods using 6 mM glutamate plus 6 mM malate as substrates; *V*_3_: state 3 respiration rate in the presence of 1 mM ADP; *V*_3_ + cyt c: state 3 respiration rate in the presence of 32 *μ*M cytochrome c. Each column represents the mean ± SEM of 4 independent experiments; ^∗^*p* < 0.05 versus control; ^#^*p* < 0.05 versus ischemia alone.

**Figure 2:**
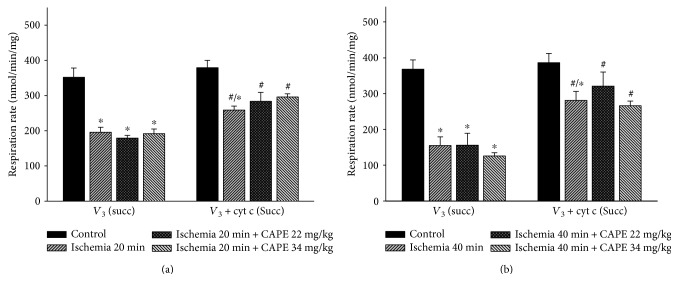
Effects of ischemia on renal mitochondrial state 3 respiration rate with succinate as substrate. Mitochondrial respiration rate was measured as described in Materials and Methods using 15 mM succinate (+2 mM amytal) as substrates. *V*_3_: state 3 respiration rate in the presence of 1 mM ADP; *V*_3_ + cyt c: state 3 respiration rate in the presence of 32 *μ*M cytochrome c. Each column represents the mean ± SEM of 4 independent experiments; ^∗^*p* < 0.05 versus control; ^#^*p* < 0.05 versus *V*_3_ of respective group.

**Figure 3:**
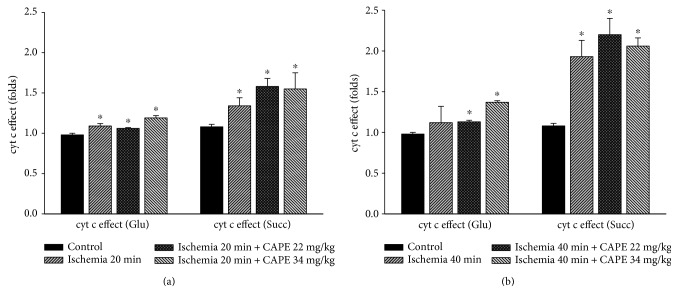
Influence of ischemia on cytochrome c effect. Cytochrome c effect was calculated as the *V*_3_ + cyt c/*V*_3_ ratio. *V*_3_ + cyt c respiration rate was measured in the presence of 1 mM ADP and 32 *μ*M cytochrome c. Each column represents the mean ± SEM of 4 independent experiments; ^∗^*p* < 0.05 versus control.

**Figure 4:**
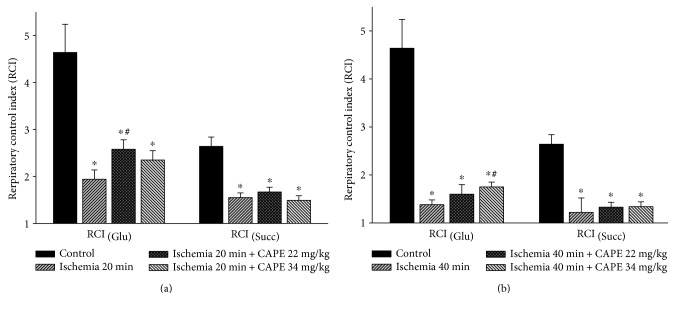
Effect of ischemia on mitochondrial respiratory control index (RCI). Measurements were performed in the presence of 5 mM glutamate + 5 mM malate or 15 mM succinate (+2 mM amytal) as substrates. Mitochondrial respiratory control index (RCI), that is, the ratio between oxygen uptake rates in state 3 and routine respiration rate (RCI = *V*_3_/*V*_0_). Each column represents the mean ± SEM of 4 independent experiments; ^∗^*p* < 0.05 versus control; ^#^*p* < 0.05 versus ischemia alone.

To investigate if CAPE protects mitochondria from ischemia-induced mitochondrial damage, short time (20 min) and longer time periods (40 min) of ischemia were chosen. As shown in Figure 1(a), ADP-dependent (state 3) respiration (*V*_3_) after 20 min of ischemia was decreased by 52% with glutamate/malate and by 44% with succinate, *p* < 0.05 (Figure 2(a)). Longer duration (40 min) of ischemia caused even greater (by 62%, Figure 1(b)) decrease of the state 3 respiration rate with glutamate/malate and succinate (decreased by 56%, Figure 2(b)). Respiratory control index (RCI) decreased by 58% and 70% with glutamate/malate as substrate and by 41% and 54% (*p* < 0.05) with succinate as substrate (Figures 3(a) and 3(b)) after 20 min and 40 min of ischemia, respectively, in accordance with the decrease in state 3 respiration rate. Leak respiration rate (i.e., without addition of ADP) remained unchanged (not shown). After addition of exogenous cytochrome c during state 3 respiration, respiratory rate (*V*_3_ + cyt c) with glutamate + malate increased by 9% and 12% and with succinate by 32% and 93% (*p* < 0.05) after 20 min and 40 min of ischemia, respectively, as compared to the control group (Figures 2(a) and 2(b)), indicating that ischemia induced damage of mitochondrial outer membrane, which increased with the duration of ischemia. The effect of cytochrome c (*V*_3_ + cyt c/*V*_3_ ratio), showing the stimulation of state 3 respiration rate after addition of cytochrome c after 20 min and 40 min of ischemia for both substrates glutamate/malate (1.09 and 1.12, resp.) and succinate (1.34 and 1.93, resp.) is shown in Figure 3. It clearly indicates the ischemia-caused damage of mitochondrial outer membrane, which was the most evidently observed in mitochondria respiring on succinate. After pretreatment of rats with two different doses (22 mg/kg and 34 mg/kg) of CAPE before 20 min of ischemia, the mitochondrial state 3 respiration (*V*_3_) increased by 20% and 19% (*p* < 0.05), respectively (Figure 1(a)), and respiratory control index (RCI) by 33% and 21% (Figure 4(a)) *p* < 0.05 with glutamate/malate as substrates. However, pretreatment of rats with the same doses of CAPE before longer time, 40 min of ischemia, had no protective effects on mitochondrial respiration rates. Moreover, CAPE had no protective effects on succinate oxidation neither after 20 min nor 40 min of ischemia (Figures 2 and 4). There was no protective effect on the mitochondrial outer membrane after pretreatment with CAPE, as the state 3 respiration rate in the presence of cytochrome c (*V*_3_ + cyt c), Figures 1 and 2, and cytochrome c effect (*V*_3_ + cyt c/*V*_3_ ratio, Figure 3) remained similar with both substrates as compared to ischemia group.

### 3.2. Effect of CAPE on Complex I Activity

**Figure 5:**
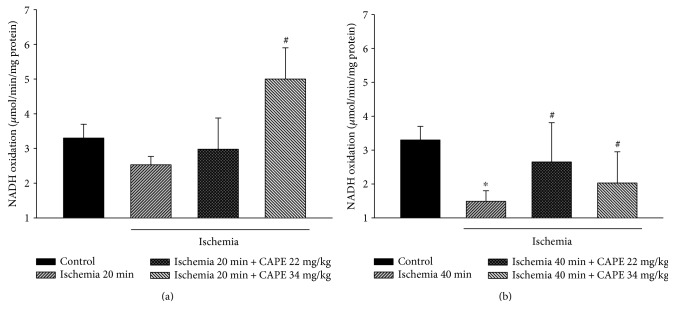
Effect of ischemia on complex I activity in kidney mitochondria. The complex I activity was measured spectrophotometrically at 340 nm as described in Materials and Methods. Each column represents the mean ± SEM of 4 independent experiments; ^∗^*p* < 0.05 versus control; ^#^*p* < 0.05 versus ischemia alone.

As our results showed that mitochondrial respiration rate with glutamate/malate (complex I-linked substrates) was clearly reduced after ischemia, in addition, we measured the effects of ischemia on mitochondrial complex I activity. Our data revealed that the reduction of state 3 respiration rate after 20 min of ischemia was associated with the decrease in complex I activity by 23% (Figure 5(a)). After 40 min of ischemia, mitochondrial complex I activity was diminished by 54% (*p* < 0.05, Figure 5(b)). Pretreatment of animals with two different doses of CAPE (22 mg/kg and 34 mg/kg) had protective effect on mitochondrial respiratory chain. After pretreatment with CAPE, complex I activity after 20 min of ischemia increased by 18% (22 mg/kg CAPE) and by 98%, *p* < 0.05 (34 mg/kg CAPE, Figure 5(a)). After 40 min of ischemia, complex I activity increased by 77%, *p* < 0.05 (22 mg/kg CAPE), and by 36%, *p* < 0.05 (34 mg/kg CAPE, Figure 5(b)).

### 3.3. CAPE Increases Mitochondrial Ca^2+^ Uptake

It is well known that mitochondria play a crucial role in intracellular Ca^2+^ signaling, taking up and releasing calcium upon different cellular conditions such as ischemia, oxidative stress, etc. Elevation of intramitochondrial calcium concentration after ischemia can trigger opening of mitochondrial permeability transition pore and cell death.

**Figure 6:**
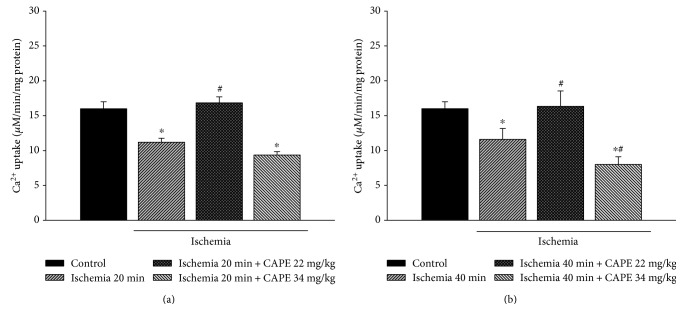
Mitochondrial Ca^2+^ uptake: effect of ischemia. Ca^2+^ uptake was measured fluorimetrically (excitation at 506 nm, emission at 535 nm) as described in Materials and Methods. Each column represents the mean ± SEM of 4 independent experiments; ^∗^*p* < 0.05 versus control; ^#^*p* < 0.05 versus ischemia alone.

Fluorimetric Ca^2+^ measurements were performed in order to measure Ca^2+^ uptake by mitochondria after 20 and 40 min of ischemia alone or after pretreatment with CAPE. Our results indicated that in control mitochondria, Ca^2+^ uptake was 15.99 *μ*mol/min mg protein. Ischemia 20 and 40 min reduced accumulation of calcium in mitochondria by 30% (Figures 6(a) and 6(b)). Pretreatment of rats with CAPE (22 mg/kg) significantly increased the mitochondrial Ca^2+^ uptake by 50% after 20 min of ischemia and by 41% after 40 min of ischemia (Figures 6(a) and 6(b)) as compared to ischemia alone. Pretreatment of animals with higher concentration of CAPE (34 mg/kg) had no protective effect on calcium accumulation in kidney mitochondria after ischemia.

### 3.4. CAPE Reduces Caspase Activation

**Figure 7:**
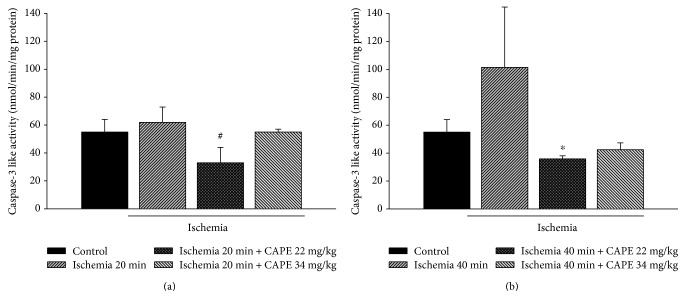
Effect of ischemia on caspase-3 activity. The caspase-3 activity was measured as described in Materials and Methods. Each column represents the mean ± SEM of 4 independent experiments; ^∗^*p* < 0.05 versus control; ^#^*p* < 0.05 versus ischemia alone.

As an indicator for apoptosis, we measured DEVD-cleaving caspase-3-like protease activity. After 20 min of ischemia, caspase-3-like activity in cytosolic fraction was increased by 1.15-fold as compared to control, whereas after pretreatment with CAPE (22 mg/kg and 34 mg/kg), caspase-3-like activity was diminished by 1.52 fold, *p* < 0.05, and returned to control level (Figure 7(a)). After 40 min of ischemia, caspase-3-like activity in cytosolic fraction was increased by 1.86-fold as compared to control. CAPE (22 mg/kg and 34 mg/kg) diminished caspase-3-like activity to control level (Figure 7(b)).

### 3.5. CAPE Reduced Lactate Dehydrogenase (LDH) Activity in Cytosolic Fraction

**Figure 8:**
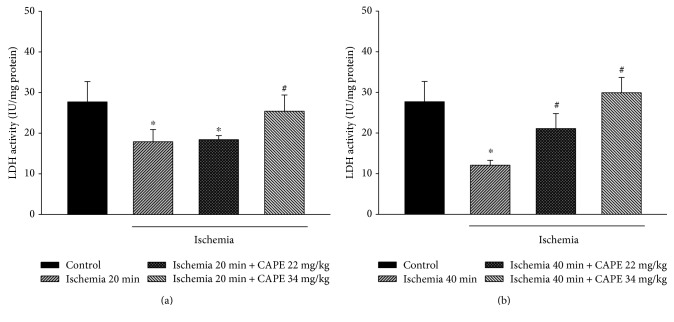
LDH activity in cytosolic fraction after ischemia. LDH activity was measured as described in Materials and Methods. Each column represents the mean ± SEM of 4 independent experiments; ^∗^*p* < 0.05 versus control; ^#^*p* < 0.05 versus ischemia alone.

As an indicator for necrosis, lactate dehydrogenase (LDH) activity was measured in cytosolic fractions in the control group and after ischemia (with and without pretreatment with CAPE). LDH activity in cytosolic fraction of control mitochondria was 27.8 ± 5.1 IU/mg protein and decreased by 35% and 56% after 20 min and 40 min of ischemia, respectively (Figures 8(a) and 8(b)). Pretreatment with CAPE (22 mg/kg) had no protective effect after 20 min of ischemia, but improved it after 40 min of ischemia (Figures 8(a) and 8(b)), that is, LDH activity in cytosolic fraction increased by 74% (to 21.1 IU/mg protein). After pretreatment with higher dose (34 mg/kg) of CAPE, activity of LDH was restored nearly to control level after both times of ischemia (Figures 8(a) and 8(b)).

### 3.6. Kidney Electron Microscopy

**Figure 9:**
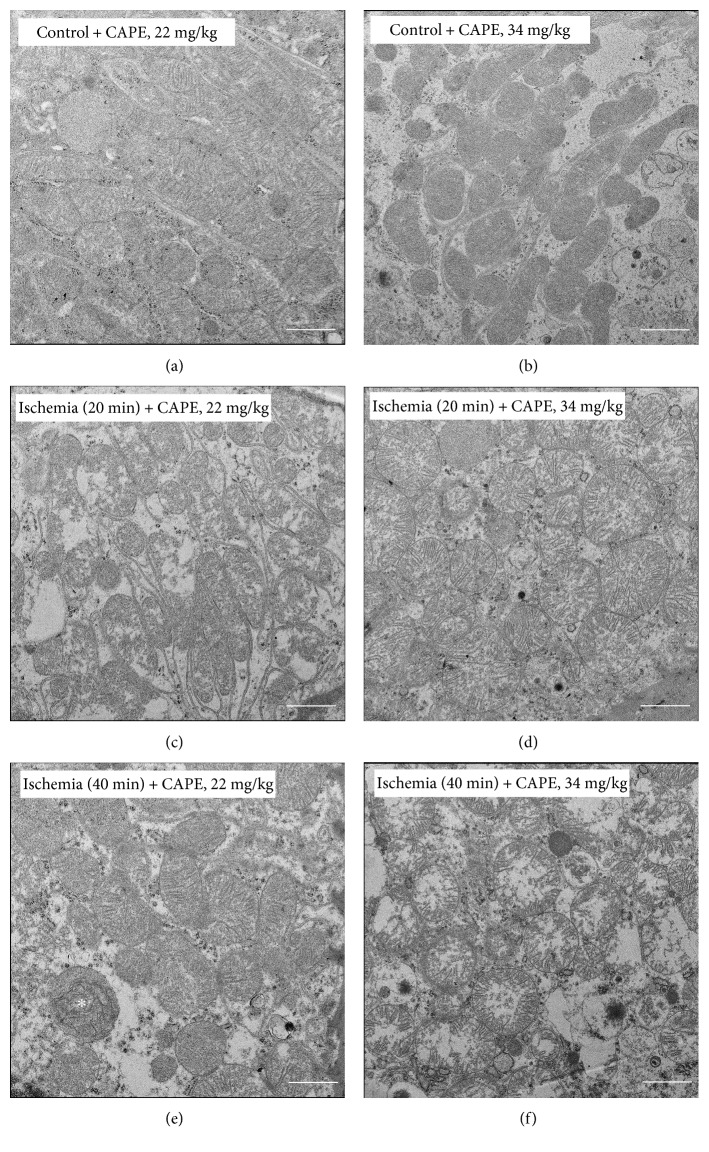
Ultrastructural changes to mitochondria. (a, b) Control. Mitochondria display normal morphology—shape is from round to elongate depending on the section, cristae are arranged in parallel rows at regular intervals, and the matrix is evenly dense. Note that normal morphology is characteristic to all mitochondria despite their irregular distribution in renal cells. (c, d) 20 min ischemia affected mitochondria: matrix is enlarged, uneven, and partly interrupted by empty patches; the outer and inner mitochondrial membranes are close and parallel. In 22 mg/kg CAPE group (c), cristae are parallel ranged; however, in CAPE 34 mg/kg group (d), irregular orientation of cristae can be seen. (e, f) 40 min ischemia affected mitochondria. (e) Cell with moderately preserved mitochondria in CAPE 22 mg/kg group: matrix is slightly enlarged with seldom empty patches, cristae are irregularly oriented, and the outer membrane is discontinuous. Note a mitochondrion with fully lost inner structure (∗). (f) Mitochondria in CAPE 34 mg/kg group possess noticeably swollen matrix and exclusively peripheral cristae, while the outer membrane is ruptured in many places. N: nucleus; RER: rough endoplasmic reticulum; Ex: extracellular space. Scale bar: 1 *μ*m.

Electron microscopical findings revealed that CAPE (22 mg/kg and 34 mg/kg) did not affect the ultrastructure of control mitochondria—they showed normal mitochondrial ultrastructure—parallel cristae, uniform matrix, and uninterrupted outer membrane. Both parts of intermembrane space—intracristal and peripheral—are narrow and even (Figures 9(a) and 9(b)). CAPE pretreatment before 20 min of ischemia affected mainly mitochondrial matrix making it slightly swollen and perforated by empty patches in some cells. Mitochondria after pretreatment with CAPE (22 mg/kg) showed less swollen matrix (Figure 9(c)) comparing with the higher CAPE concentration (34 mg/kg) (Figure 9(d)). Following enlarged matrix cristae lose their parallel arrangement and rearrange to radial location filling almost all volume of mitochondria (Figure 9(d)). After 40 min of ischemia, mitochondria increased in size due to enlarged amount of matrix; their cristae lost parallel arrangement. Seldom, mitochondria were seen broken (Figures 9(e) and 9(f)). However, CAPE (22 mg/kg) preserved continuous matrix with sporadically seen small patches and only partly lost cristae parallelism (Figure 9(e)). Higher dose of CAPE (34 mg/kg) preserved ultrastructure of mitochondria; less and swollen matrix with disintegrated cristae and ruptured outer membrane were usually observed (Figure 9(f)). Intermembrane space in all experimental groups was narrow and even.

## 4. Discussion

Mitochondria play an important role in the pathogenesis of ischemic kidney injury as they are responsible for more than 90 percent energy production by oxidative phosphorylation [16]. Therefore, the decrease in mitochondrial function may lead to renal dysfunction and cell death. The protective substances against ischemic kidney injury especially when prolonged time of ischemia during kidney surgery is necessary are of great importance.

In this study, we investigated if CAPE has potential protective effects against short (20 min) and longer time (40 min) ischemia-induced kidney damage in an *in vitro* rat model of warm kidney ischemia. We measured mitochondrial functions, mitochondrial calcium uptake, caspase-3 activation, and lactate dehydrogenase amount. Our novel finding is that CAPE ameliorates in part ischemia (20 min)-induced renal mitochondrial injury in rats, improves oxidative phosphorylation with complex I-dependent substrate glutamate/malate, increases Ca^2+^ uptake by mitochondria, partially blocks ischemia-induced caspase-3 activation, and protects kidney cells from ischemia-induced necrosis. Thus, a single intraperitoneal injection of CAPE (22 mg/kg and 34 mg/kg) 1.5 hour before ischemia partially protects mitochondria from injury caused by ischemia. The protective effects on mitochondrial respiration rates were seen after shorter (20 min) time of ischemia whereas reduction of apoptosis and necrosis and increase in Ca^2+^ uptake were revealed after both, shorter (20 min) and longer (40 min) time of ischemia.

The model of 20 and 40 min kidney ischemia *in vitro* was chosen because, according to our results [15], 20 min was the shortest period of ischemia that induced statistically significant changes in renal mitochondrial respiratory functions. Moreover, our results showed that 40 min of kidney ischemia induced much more severe mitochondria structural and metabolic changes. The dose of CAPE (22 mg/kg or 34 mg/kg) was chosen based on the reported doses (intraperitoneal or intravenous) in the literature, ranging between from 1 mg/kg to 40 mg/kg [12, 13, 17]. They showed that CAPE at abovementioned doses ameliorates oxidative damage caused by ischemia/reperfusion in brain, or intestine tissue [12, 13, 17]. According to literature data, CAPE distributes into tissues extensively [18] and the elimination half-life is ranging between 21.2–26.7 min [18] after intravenous administration.

Ince et al. described cardioprotective effects of CAPE in short time ischemia (8 min)-reperfusion (8 min) model of rats. The protective effects are explained by decreased activity of xanthine oxidase and direct antioxidant effects [19]. Other authors [20–24] showed the protective effects of CAPE against oxidative stress-induced kidney injury. CAPE exerts antioxidant activity by suppressing lipid peroxidation, scavenging ROS, and inhibiting activity of nitric oxide synthase and xanthine oxidase [25]. It has been shown [26] that CAPE at concentration of 10 *μ*M inhibited the xanthine/xanthine oxidase system in human neutrophils and decreased production of reactive oxygen species. It is known that CAPE is a lipophilic compound and may interact with phospholipid bilayers of membranes, including mitochondria, and in this way might protect cell organelles from ROS-induced damage. Phenethyl group enhanced the radical scavenging capacity of CAPE as esterification of phenolic acids increases lipophilicity, and they can better incorporate into membranes. The antiradical activities of CAPE are explained by ortho-dihydroxyl functionality in the catechol ring [27]. So, we hypothesize that at least a partial protective effects against oxidative stress-induced tissue damage may be related to CAPE action at the mitochondrial level. However, most of the studies were done by investigating the markers of oxidative stress, and there is a lack of data on mitochondrial status. There are only a few studies regarding CAPE effects on mitochondria. Recently, Kobroob et al. [23] showed that CAPE restored the decline in mitochondrial membrane potential in renal mitochondria during oxidative stress caused by cadmium and attenuated cadmium-induced swelling of mitochondria. Moreover, another study [28] revealed that CAPE and its related compounds protect mouse brain and liver mitochondria from damage during *in vitro* anoxia-reoxygenation. Thus, our study revealed a partial increase in mitochondrial oxidative phosphorylation capacity with NAD-linked substrate glutamate/malate after administration of CAPE prior induction of short (20 min) ischemia. We also showed that the ischemia-diminished activity of complex I was also protected after CAPE injection. As complex II—dependent substrate—succinate oxidation was not improved by CAPE, we conclude that CAPE has specific action on complex I.

Feng et al. revealed that the protective effects of CAPE are due to limiting of mitochondrial membrane lipoperoxidation, membrane fluidity, and protein carbonylation in anoxia-reoxygenation, which resulted in the maintenance of mitochondrial function [28]. Our results also revealed the restoration of mitochondrial functions (a partial increase in the state 3 respiration rate and in a respiratory control index) after CAPE pretreatment before 20 min of ischemia. However, pretreatments of rats with CAPE before induction of severe 40 min ischemia had no protective effects on mitochondrial respiration rates, but it had positive effects on reduction of apoptosis, necrosis, and Ca^2+^ uptake by mitochondria.

It is well known that mitochondria play a crucial role in intracellular Ca^2+^ signaling. Mitochondria can transiently accumulate large amounts of Ca^2+^ if cytosolic Ca^2+^ concentration increases as response to ischemia, stress, or other pathological conditions. They can locally buffer Ca^2+^ modulating the activity of Ca^2+^ channels if there is Ca^2+^ influx through the plasma membrane. In mitochondria, Ca^2+^ at physiological concentrations can regulate mitochondrial energy metabolism whereas calcium overload can induce the release of cytochrome c and activation of apoptotic cell death [29]. Our results indicated that in kidney mitochondria, ischemia (20 and 40 min) induced decreases in oxidative phosphorylation and subsequent Ca^2+^ uptake. It is important to note that mitochondrial Ca^2+^ uptake after ischemia (20 min and 40 min) was clearly improved after administration of CAPE (22 mg/kg). The increased capacity of mitochondria to uptake calcium may be due to increase in oxidative phosphorylation capacity caused by CAPE.

Moreover, we also revealed that increase in caspase-3 activation during ischemia was blocked by CAPE (22 mg/kg). These results are in line with the observation of Tan et al. who showed that in heart mitochondria, CAPE (3 mg/kg), injected 60 min before ischemia, protects from calcium-induced caspase-3 activation [30]. Thus, CAPE, depending on concentration, has a protective effect from caspase activation. Moreover, our results showed that not only apoptosis but also necrosis occurs after ischemia: as LDH changes in cytosolic fraction after both time of ischemia as compared to control, it shows the signs of necrosis as well. It is possible that both, apoptosis and necrosis, occur in ischemic kidney. Our results revealed that both used concentrations of CAPE-reduced LDH release and diminished necrosis.

Ultrastructural investigation revealed that CAPE had protected from ischemia (20 min)-induced mitochondrial damage. Previously, we have demonstrated that ischemia mainly affects the mitochondrial intermembrane space, where intracristal space was found enlarged resulting in ballooned cristae as well as enlarged peripheral space followed by detachment of outer and inner membranes [15]. Application of CAPE preserved intermembrane space from edema after 20 min of ischemia, as it was found narrow and uniform in all experimental groups. No ballooned cristae, with enlarged intracristal space or detachment of outer and inner membranes resulting in enlarged peripheral space, were observed in this study. Though pretreatment with the higher dose (34 mg/kg) of CAPE protected slightly mitochondrial functions (oxidative phosphorylation, activity of mitochondrial complex I, LDH), whereas ultrastructure of mitochondria in some kidney tissue slices showed swollen matrix with disintegrated cristae and ruptured outer mitochondrial membrane, thus it seems that higher dose of CAPE did not protect ultrastructure of mitochondria. The obtained effects may be associated with the heterogeneity of mitochondria. Our observations suggest that different renal cell types are affected by ischemia to a different extent. Thus, it would be feasible to investigate the protective properties of CAPE in different renal cell types.

In conclusion, our study revealed partial protection of mitochondrial function after pretreatment of rats with intraperitoneal injection of CAPE. Since CAPE has beneficial, including antioxidant and anti-inflammatory, effects as well as partially preserves mitochondrial function, we think that this compound may have a potential to protect the kidney from ischemia-induced damage. The mechanisms of protection are not fully understood yet, but at least, partially, it is associated with the improvement of mitochondrial status in the cells. Further studies will be required for the better characterization of the mechanism of CAPE action.
